# Intranasal Insulin Administration Does Not Affect LH Concentrations in Men with Diabetes

**DOI:** 10.1155/2018/6170154

**Published:** 2018-10-31

**Authors:** Sandeep Dhindsa, Rama Chemitiganti, Husam Ghanim, Evangelina Santiago, Adnan Haider, Natalia Chaar, Mary Mok, Alexis McKee, Paresh Dandona

**Affiliations:** ^1^Division of Endocrinology, Diabetes and Metabolism, Texas Tech University Health Sciences Center, 800 West 4th Street, Odessa, TX 79763, USA; ^2^Division of Endocrinology, Diabetes and Metabolism, State University of New York, Buffalo and Kaleida Health 462 Grider Street, Buffalo NY-14215, USA; ^3^Division of Endocrinology, Diabetes and Metabolism, Saint Louis University, 1402 S Grand Blvd, St. Louis MO-63141, USA

## Abstract

A quarter of men with obesity or type 2 diabetes have hypogonadotropic hypogonadism. Animal studies and in vitro data have shown that insulin action and insulin responsiveness in the brain are necessary for the maintenance of the functional integrity of the hypothalamo-hypophyseal-gonadal axis. We conducted a randomized, placebo-controlled trial to evaluate the effect of one dose of intranasal insulin (40 IU of regular insulin) or saline on LH concentrations in 14 men (8 with type 2 diabetes and 6 healthy lean men). Insulin or saline was administered intranasally on two different occasions, at least one week apart. Blood samples were collected to measure LH concentrations every 15 minutes for 5 hours. Study drug was administered intranasally after a 2-hour baseline sampling period. Patients remained fasting throughout the procedure. The primary endpoint of the study was to compare the change in LH concentrations after intranasal insulin as compared to placebo (intranasal saline). Change was defined as the difference between baseline LH concentrations (average of the 9 samples collected in two hours prior to drug administration) and average LH concentrations following drug administration (average of the 12 samples collected in 3 hours). There was no change in LH concentrations following insulin administration as compared to placebo in men with diabetes or in lean men. We conclude that one dose of 40 IU of regular insulin administered intranasally does not change LH concentrations acutely in men.

## 1. Introduction

One-third of men with type 2 diabetes have subnormal free testosterone concentrations in association with inappropriately low LH and FSH concentrations [1]. Magnetic resonance imaging in these hypogonadal patients showed no abnormality in the brain or the pituitary [1]. The response of LH and FSH to GnRH injection is also normal. In addition, approximately 25% of obese nondiabetic men and men with metabolic syndrome also have hypogonadotropic hypogonadism (HH). The prevalence of subnormal testosterone concentrations is directly proportional to obesity in these men [2]. This association is observed at all ages, including in young men and obese adolescents [3].

The cause of HH observed in association with insulin resistance is not well understood. Since T and androstenedione in the male can be converted to estradiol and estrone, respectively, through the action of aromatase in the mesenchymal cells and preadipocytes of adipose tissue, it has been suggested that excessive estrogen secretion due to aromatase activity in the obese may potentially suppress the hypothalamic secretion of gonadotropin-releasing hormone (GnRH) [4, 5]. Estradiol concentrations are elevated in obese men [6]. It therefore follows that the estradiol concentrations in men with HH and obesity should be elevated to account for the suppression of gonadotropin secretion. However, estradiol concentrations are lower in males with HH as compared to eugonadal obese males [7, 8]. This is true in men at all ages (adolescents, middle-age, and elderly) [7, 9, 10]. Thus, other factors associated with obesity likely account for the HH. It is now known that kisspeptin, a hypothalamic neuropeptide encoded by the KISS1 gene and the presence of kisspeptin receptors on the GnRH neurons (G protein-coupled receptor 54), is obligated for the release of GnRH. Humans with absence of either kisspeptin gene or its receptor (GPR54) have HH [11, 12]. Intravenous administration of kisspeptin increases LH and testosterone concentrations in men with type 2 diabetes and HH [13], thus suggesting that the hypothalamic-pituitary-gonadal axis is intact per se in men with HH and type 2 diabetes. However, the (presumably) metabolic insult in insulin resistance that results in hypogonadotropism is yet to be defined. Kisspeptin neurons express both leptin and insulin receptors, thus possibly accumulating evidence of metabolic health and translating it into reproductive health. Leptin appears to serve as a signal of energy reserves to regulate the hypothalamo-pituitary-gonadal axis in relation to nutritional status [14]. It is possible that leptin resistance in neurons may contribute to the pathogenesis of hypogonadotropism seen in obesity. Direct evidence in humans supporting or disproving this reasoning is, however, lacking. Interestingly, the selective deletion of the insulin receptor from neurons leads to a reduction in LH and T concentrations by 60%–90% and low T concentrations [15]. These animals respond to GnRH challenge by normal or supranormal release of LH. In addition, it is known that the incubation of hypothalamic neurons with insulin results in the facilitation of secretion of GnRH [16, 17]. Thus, insulin action and insulin responsiveness in the brain are necessary for the maintenance of the functional integrity of the hypothalamo-hypophyseal-gonadal axis. We therefore attempted to evaluate the effect of intranasal insulin on LH concentrations in men with type 2 diabetes. We hypothesized that one dose of 40 IU of insulin administered intranasally will lead to an increase in LH concentrations. We chose 40 IU dose because this dose has been shown to increase cerebral blood flow in patients with diabetes [18].

## 2. Materials and Methods

This was a prospective, randomized, double-blind, placebo-controlled, crossover, single-center, proof-of-concept study to assess the effect of one dose of intranasal insulin or saline on LH concentrations. The trial was conducted at Permian Basin campus of Texas Tech University Health Sciences Center (Odessa, TX). The protocol was approved by the institutional review board of Texas Tech University Health Sciences Center. An informed consent was signed by all subjects. The trial was registered with ClinicalTrials.gov (NCT 02154477).

### 2.1. Study Population

Eight with type 2 diabetes were recruited for the study from endocrinology clinic of Texas Tech University physician group practices in the Permian Basin between March 2015 and October 2016. Subjects were between 18 and 75 years of age with HbA1c < 8.5%. Subjects on androgens, glucocorticoids, or opiates in the last 6 months and subjects with panhypopituitarism, severe hepatic or kidney disease (glomerular filtration rate < 30 ml/min/m^2^), HIV, hepatitis C, untreated severe obstructive sleep apnea, type 1 diabetes, or suffering from a chronic infectious or inflammatory condition were excluded from the study.

We recruited 6 lean (BMI < 25 kg/m^2^) healthy men to serve as a comparison group. These men did not have any diagnosed health conditions and were not on any medications.

### 2.2. Study Design

Subjects who qualified for the study were asked to come in a fasting state for 2 study visits, at least one week apart. Subjects in the diabetes group were asked not to take their oral hypoglycemic or insulin in the morning of the study visit. A peripheral intravenous cannula was placed for blood draws. Since LH is secreted in a pulsatile manner (at the rate of ~0.8 pulses/h) LH concentrations, we collected blood samples at 15-minute intervals throughout the study [19]. Study drug was administered intranasally after a 2-hour baseline sampling period.

### 2.3. Study Drug Administration

40 IU of regular insulin or equivalent volume of normal saline was administered intranasally with ViaNase device on two different occasions, at least one week apart. ViaNase is a liquid drug delivery system based on controlled particle dispersion technology. It is manufactured by Kurve Technology (http://www.kurvetech.com). The device was donated by Kurve Technology for this research project. The device has a disposable nose piece and hence can be reused. The insulin (Humulin R, 100 IU/ml) was reconstituted for each application. The device delivers a metered dose of insulin into the chamber that covers the subject's nose. Using the principal of vortical flow, controlled particle dispersion effectively disrupts inherent nasal cavity airflows to deliver formulations to the olfactory region and the paranasal sinuses while minimizing peripheral deposition to the lungs and stomach. It is assumed that after intranasal administration, insulin travels extracellularly through patent intercellular clefts in the olfactory epithelium to diffuse into the subarachnoid space [20]. It has been shown that 40 IU of intranasal insulin administration results in a rapid increase in cerebrospinal fluid insulin concentrations by 80% for one hour [21]. There is no change in serum insulin concentrations.

Blood samples to measure LH concentrations were collected every 15 minutes for 3 hours following the study drug administration. Thus, the total duration of each study visit was 5 hours. Blood glucose was measured prior to the administration of the study drug and 30, 60, and 180 min thereafter.

### 2.4. Laboratory Measurements

Serum LH concentrations were measured by chemiluminescent immunometric assay at a commercial laboratory (LabCorp). Total and free testosterone concentrations were measured at baseline by liquid chromatography tandem mass spectrometry (LC-MS/MS) and equilibrium dialysis at a commercial laboratory (Nichols Institute, Chantilly, VA, Quest Diagnostics) as previously described [22]. The sensitivity of the assay (LOQ), set at a coefficient of variation (CV) of ≤20%, was 0.01 nmol/l. The intra-assay CV ranged from 7.6 to 10.8% and interassay CV ranged from 9.8 to 13.4% at total testosterone concentrations between 0.34 and 41.7 nmol/l. Reference range for total testosterone (8.7–38.2 nmol/l) was determined from 264 apparently healthy men. As per the measuring laboratory, free testosterone concentration below 0.174 nmol/l was defined as subnormal.

### 2.5. Statistical Analysis

The primary endpoint of the study was to compare the change in LH concentrations after intranasal insulin as compared to placebo (intranasal saline) by *t*-test. Change was defined as the difference between baseline LH concentrations (average of the 9 samples collected in two hours prior to drug administration) and average LH concentrations following drug administration (average of the 12 samples collected in 3 hours). Type I error (*α*) was set at 0.05 and type II error (*β*) at 0.2. We hypothesized that there will be a 50% increase in LH concentrations. A convenience sample size of 8 men with type 2 diabetes and 6 healthy lean men was taken to test the hypothesis in this proof of concept study. All data were normally distributed and are presented as means ± SD (or means ± SE where indicated). The SPSS software (SPSS Inc., Chicago, Illinois) was used for analysis.

## 3. Results

### 3.1. Baseline Characteristics of Men with Diabetes

The mean age and BMI of men with type 2 diabetes were 63 ± 7 years (range 51–72 years) and 34 ± 5 kg/m^2^ (range 25–40 kg/m^2^), respectively. All men had hypertension and 3 subjects had coronary artery disease. Three subjects had microvascular complications (two subjects had albuminuria and one had retinopathy). Mean HbA1c was 7.3 ± 0.8%. All men were on oral hypoglycemic medications: sulfonylurea (2 men), metformin (all men), pioglitazone (2 men), and empagliflozin (1 man). In addition, 4 men were also taking insulin.

The mean total and free testosterone concentrations were 12.8 ± 6.1 and 0.194 ± 0.066 nmol/l, respectively. Two men with diabetes had subnormal free and total testosterone concentrations. Both these men had normal baseline LH concentrations. Thus, they had HH. One man had supranormal LH (15 IU/l) and normal free testosterone concentration. Thus, he had compensated hypogonadism. He had normal testicular size (15 ml) and did not have a history of testicular trauma, orchitis, or infertility. One man had subnormal total testosterone but normal free testosterone.

### 3.2. Baseline Characteristics of Lean Men

The mean age and BMI of subjects in the lean group were 30 ± 9 years (range 23–42 years) and 22 ± 2 kg/m^2^ (range 20–24 kg/m^2^), respectively. All men in the lean group had normal total and free testosterone concentrations. The mean total and free testosterone concentrations were 22.6 ± 8.3 and 0.358 ± 0.285 nmol/l, respectively, in lean men.

The mean age and BMI of men with type 2 diabetes were higher than those for lean men (*p* < 0.001), while their total and free testosterone concentrations were lower (*p* = 0.05 and 0.001, respectively). The mean total and free testosterone concentrations remained lower in men with diabetes (*p* = 0.05 and 0.03, respectively) after adjustment for age and BMI. Baseline LH concentrations were higher in men with diabetes (7.8 ± 3.5 IU/l) than in lean men (3.6 ± 0.9 IU/l, *p* = 0.01). However, the mean LH concentrations in lean men and men with diabetes were similar after adjusting for age and BMI (5.0 and 6.8 IU/l, respectively, *p* = 0.70).

### 3.3. Change in LH Concentrations during the Study

**Figure 1:**
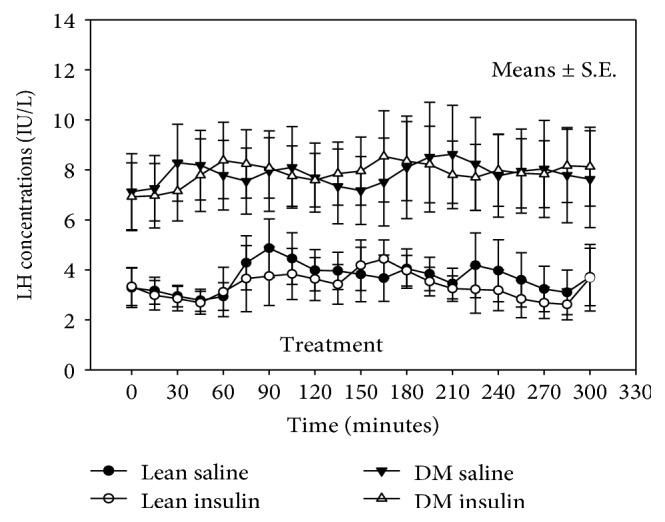
LH concentrations during the study in lean men and men with diabetes mellitus (DM).

The mean LH concentrations did not change after intranasal insulin in healthy lean men or in men with diabetes (Figure 1 and Tables 1 and 2). The LH pulse frequency or peak LH concentration also did not change in either group after intranasal insulin.

There was no change in LH concentrations after excluding the 2 men with HH in the diabetes group (−0.2, [−1.8, 1.5], *p* = 0.88). LH concentrations did not change after intranasal insulin therapy when men on subcutaneous insulin were excluded from the analysis (−0.05, [−1.3, 1.2], *p* = 0.92). There was no change in LH concentrations after excluding the man with elevated LH concentrations (−0.3, [−2.0, 1.4], *p* = 0.70).

The baseline LH concentrations were not related to change in LH concentrations after intranasal insulin in lean mean (*r* = −0.47, *p* = 0.35) or in men with diabetes (*r* = 0.46, *p* = 0.25).

The glucose concentrations after insulin administration (average of measurements at 30, 60, and 120 minutes) did not change as compared to baseline in lean men or in those with diabetes (Tables 1 and 2). There was a decrease in blood sugars while fasting in men with diabetes during both insulin and saline visits.

## 4. Discussion

Our data show that one dose of 40 IU of regular insulin administered intranasally does not change LH concentrations acutely in healthy lean men or in men with diabetes. There was no change in LH pulsatility or maximum LH concentration either. The lack of effect of intranasal insulin is lean men was not entirely surprising since they are not insulin resistant and presumably had normal insulin signaling in the central nervous system. Our results are consistent with another experiment of intranasal insulin in lean men that measured LH, FSH, and testosterone concentrations every 30 minute for 2 hours [23]. There was no change in LH, FSH, or testosterone concentrations after 40, 80, or 160 IU of intranasal insulin administration. Another trial in healthy men also did not show a change in fasting LH, FSH, or testosterone concentrations after 4 weeks of treatment with intranasal insulin, 40 IU, 4 times a day [24]. Ours is the first study to assess the effect of intranasal insulin on LH concentrations in men with type 2 diabetes, a population that has insulin resistance and a high prevalence of HH.

Our study was based on the assumption that insulin resistance in the central nervous system is, at least partly, responsible for hypogonadotropic hypogonadism observed in men with diabetes, and that intranasal insulin may restore insulin signaling. Almost all the GnRH neurons and 5–20% of kisspeptin neurons in mice express insulin receptors [25]. Animal studies have shown that insulin signaling in neurons is necessary for GnRH release. However, it is unlikely that insulin acts directly on GnRH neuron. Isolated knockout of insulin receptor in GnRH neuron does not lead to a decrease in LH concentrations or in fertility in either male or female mice [26]. The loss of insulin receptors in proopiomelanocortin- or agouti-related peptide-expressing (POMC- or AgRP-expressing) neurons—neurons involved in the regulation of appetite and peripheral metabolism of glucose and fat—also did not affect fertility [27]. Neither does the selective deletion of insulin receptors from kisspeptin neurons affect LH concentrations, T concentrations, and fertility in mice [28]. Thus, the site (or sites) of hypogonadotropism seen in neuronal insulin receptor knockout mice is not clear. Insulin signaling in the brain is widespread [29], and prevention of insulin signaling in one type of neuron may not be enough to disrupt the hypothalamo-pituitary-gonadal axis.

Over recent years, there has been a greater appreciation of the effects of insulin in the central nervous system. Havrankova et al. and Sara et al. showed in 1978 that insulin receptors are present throughout the rat CNS, followed closely by the demonstration that insulin receptors are also expressed in the human brain [30, 31]. The insulin receptor is found in particularly high densities in brain regions like the olfactory bulb, the cerebellum, the dentate gyrus, the pyriform cortex, the hippocampus, the choroid plexus, and the arcuate nucleus of the hypothalamus. It is assumed that peripheral insulin crosses the blood-brain barrier by a saturable, receptor-mediated transport mechanism and by binding to brain insulin receptors and affects functions as diverse as energy and glucose homeostasis, reproduction, and cognition [15, 18, 32]. There are good data in rodents that insulin signaling in the central nervous system suppresses hepatic glucose output [33]. Lack of neuronal insulin receptors leads to mild diabetic phenotype in mice, as well as hypogonadotropic hypogonadism as mentioned above [15]. Studies also suggest that intranasal insulin reduces sympathetic outflow to adipose tissue and decreases free fatty acid concentrations in mice [34]. There are only a few human studies on intranasal insulin's effects on metabolism. Acute administration of intranasal insulin reduces food intake and chronic administration reduces fat mass [35, 36]. Recent studies show that 160 IU of intranasal insulin reduces free fatty acids and increases insulin sensitivity in lean men [37–39]. There is a reduction in hepatic glucose output as well as increased peripheral glucose uptake [38]. This is accompanied by an increase in activity of the parasympathetic nervous system and changes in cerebral blood flow and activity in the hypothalamus and striatum (as assessed by functional magnetic resonance imaging). However, there was no change in insulin sensitivity, free fatty acid concentrations, or striatal brain activity of overweight/obese men [38, 40]. Presumably, there is “central insulin resistance” to these effects in obese men [41, 42] and even 160 IU of intranasal insulin cannot overcome the central resistance. In contrast, a single dose 40 IU of intranasal insulin in patients with diabetes was found to improve cerebral blood flow and visuospatial memory [18]. Intranasal insulin at a low dose (20–40 IU daily for 4 months) has also been shown to improve memory and cognition in patients with Alzheimer's disease [43].

We did not notice a change in serum glucose concentrations. Thus, it is unlikely that there was a significant absorption of intranasal insulin into the peripheral circulation. This is consistent with prior studies [18, 21]. We avoided patients with uncontrolled diabetes (A1c > 8.5%). Prior studies have shown that hyperglycemia may decrease LH pulsatility in type 1 and type 2 diabetic men [44, 45]. However, HbA1c does not seem to impact serum testosterone concentrations [1].

Our study has several limitations. Due to lack of effect on any LH parameter after intranasal insulin, we did not conduct detailed deconvolutional analysis of LH pulsatility. Based on prior studies, we have assumed that 40 IU on intranasal insulin results in a significant increase in insulin concentrations in the central nervous system. However, we did not obtain cerebrospinal fluid to measure the insulin concentrations. Secondly, we do not know if the dose of 40 IU is adequate to overcome the presumed central insulin resistance. Lastly, we tried a single administration of insulin. It is possible that repeated stimuli of insulin over days, or a higher dose, may be necessary for the hypothalamo-pituitary-gonadal axis to resume normal production of LH in men with HH.

## 5. Conclusion

One dose of 40 IU of regular insulin administered intranasally does not change LH concentrations acutely in either healthy lean men or in men with diabetes.

## Figures and Tables

**Table 1:** LH concentrations before and after intranasal insulin or saline administration in men with diabetes.

Diabetes	Insulin			Saline			Mean difference (95% confidence interval)	*p*
	Baseline (0–120 minutes)	Posttreatment (135–300 minutes)	*p*	Mean baseline LH (0–120 minutes)	Mean LH after treatment (135–300 minutes)	*p*		
Mean LH (IU/l)	7.7 ± 3.0	8.0 ± 3.4	0.17	7.8 ± 3.5	7.9 ± 4.3	0.86	0.3, [−1.2, 1.7]	0.70
Peak LH (IU/l)	9.2 ± 3.3	9.5 ± 3.7	0.33	9.2 ± 3.7	9.8 ± 5.0	0.50	−0.2, [−2.4, 2.0]	0.87
Number of LH pulses (per hour)	0.5 ± 0.3	0.5 ± 0.4	0.74	0.6 ± 0.3	0.3 ± 0.2	0.06	0.3, [−0.1, 0.6]	0.10
Glucose (mmol/l)	8.1 ± 1.6	7.6 ± 1.7	0.01	7.5 ± 1.2	6.7 ± 1.4	0.06	0.3, [−0.5, 1]	0.45

The penultimate column shows the mean difference between the two treatments.

**Table 2:** LH concentrations before and after intranasal insulin or saline administration in healthy lean men.

Lean	Insulin			Saline			Mean difference (95% confidence interval)	*p*
	Baseline (0–120 minutes)	Posttreatment (135–300 minutes)	*p*	Mean baseline LH (0–120 minutes)	Mean LH after treatment (135–300 minutes)	*p*		
Mean LH (IU/l)	3.3 ± 1.4	3.4 ± 1.2	0.76	3.6 ± 0.9	3.8 ± 1.4	0.63	−0.1, [−1.1, 0.9]	0.83
Peak LH (IU/l)	4.6 ± 2.0	5.0 ± 1.4	0.51	5.3 ± 1.8	5.4 ± 1.9	0.92	−0.1, [−1.7, 1.5]	0.89
Number of LH pulses (per hour)	0.4 ± 0.2	0.4 ± 0.4	0.87	0.3 ± 0.3	0.3 ± 0.4	0.99	0.0, [−0.5, 0.5]	0.90
Glucose (mmol/l)	5.2 ± 0.7	4.9 ± 0.4	0.37	5.2 ± 0.5	5.1 ± 0.2	0.41	−0.1, [−1, 0.8]	0.74

The penultimate column shows the mean difference between the two treatments.

## Data Availability

The data used to support the findings of this study are available from the corresponding author upon request.
